# Difficulties in Performing Daily Activities in Patients with Dry Eye before and after Treatment

**DOI:** 10.3390/medicina59010025

**Published:** 2022-12-23

**Authors:** Gabriel Martinescu, Camelia Margareta Bogdanici, Irina Andreea Pavel, Manuela Ciocoiu

**Affiliations:** 1 Department of Ophthalmology, Faculty of Medicine, University of Medicine and Pharmacy “Grigore T. Popa”, University Street, No. 16, 700115 Iasi, Romania; 2 Department of Pathophisiology, Faculty of Medicine, University of Medicine and Pharmacy “Grigore T. Popa”, University Street, No. 16, 700115 Iasi, Romania

**Keywords:** dry eye disease, quality of life, questionnaire, daily activities, treatment

## Abstract

Dry eye disease (DED) represents an important public health problem causing visual discomfort which affects the quality of life. This paper investigates the current comprehension of DED on life quality and vision. Methods: This research consists of a cross-sectional study of 121 patients, with a mean age of 70 ± 9 years, diagnosed with DED. All patients were treated in the University Clinic for Ophthalmology in “St. Spiridon” Emergency Hospital, Iasi. For all patients, we evaluated visual acuity on the Snellen chart, tear breakup time (TBUT), Schirmer I test scores, and contrast sensitivity. For this study, we used the Visual Functioning Questionnaire—25 (VFQ-25) version 2000, modified and adapted for this research (19 items). Results: Prior to treatment, patients had very high difficulty reading a text in a newspaper or on TV, reading prices on products in shops, or recognizing people they already met. Performing manual work or favorite activities was also very difficult. Post-treatment visual challenges improved in the majority of cases, regardless of the treatment method used. Conclusions: We found that symptomatic dry eye disease was associated with reduced ability in performing several important vision-related daily tasks and has a significant impact on life quality and visual performance.

## 1. Introduction

The quality of life is based on an individual’s perception of different kinds of social situations, on the cultural value systems in which they live, and in relation to their own needs, standards, and aspirations. To be more specific, the quality of life in medicine means physical, mental, and social well-being and the ability to be independent and carry out usual tasks in their daily lives.

Global prevalence of dry eye disease is approximately 11.59% [1]. However, it varies significantly from 6.8% to 35.4% or 57.7% in different races and geographical regions. A study conducted in China on 696 patients showed that the prevalence of DED in Mongolian and Han ethnicities was 32.6% and 35.4%, respectively [2]. Another study conducted on Mediterranean Caucasian population found a prevalence of DED of 57.7% among the participants [3]. Current studies estimate that approximately 6.8% of the adult US population have been diagnosed with dry eye disease [4]. The prevalence of dry eye disease is higher among women when compared to men and increases with age [5].

Dry eye disease is a chronic multifactorial condition of the ocular surface characterized by failure to produce high quality or sufficient amounts of tears. Although 10% of individuals have aqueous deficient dry eye disease, more than 80% have either the hyperevaporative type related to meibomian gland dysfunction or a combination of both [6].

The Dry Eye WorkShop II (DEWS II) from the Tear Film and Ocular Surface Society (TFOS) defines DED as a multifactorial disease of the ocular surface characterized by loss of homeostasis of tear film, accompanied by ophthalmological symptoms, in which tear film instability and hyperosmolarity, eye surface inflammation and damage, and neurosensory abnormalities play key roles. The DEWS II study focuses more on the underlying pathogenesis of DED, including inflammation, hyperosmolarity of tears, neurosensory modifications, and unstable tear films [7].

When changes in the tear film layers occur, signs and symptoms are usually observed by patients. Presenting symptoms include itching, watering, eye dryness, irritation, eye fatigue, a sensation of grittiness, burning or soreness, and redness. Patients may also report vision changes, photophobia, trouble driving at night, discomfort while watching television or reading, itching, increased blinking, or contact lens intolerance. Unmanaged DED diminishes the quality of life, impairing activities such as prolonged reading, driving, or computer use [8,9,10,11].

Although the ideal treatment has not yet been established, the optimal therapy for DED is associated with improved bioavailability, minimal ocular side effects, and effective dosing [12].

The purpose of this study is to analyze the challenges of adjusting to daily activities of patients with dry eye syndrome before and after receiving various treatment techniques.

## 2. Materials and Methods

The study took place at the University Clinic of Ophthalmology of “St. Spiridon” Emergency Hospital, Iasi, between March 2018 and October 2019. The research was conducted respecting the Declaration of Helsinki guidelines (approval number 720/3.10.2017 for clinical study of “St. Spiridon” Emergency Hospital, Iasi). Each patient read and signed the informed consent to be part of this research.

This paper is a cross-sectional study of 121 patients, with a mean age of 70 ± 9 years, diagnosed with DED. A total of 49 patients received invasive procedures (temporary or permanent occlusion of the lacrimal punctum or temporary tarsorrhaphy) and drug treatment and 72 patients only received drug treatment.

We evaluated visual acuity on the Snellen chart, tear breakup time (TBUT), Schirmer I test scores, and contrast sensitivity in all patients. 

The TBUT is the interval between the last blink and the appearance of the first randomly distributed dry spot. A TBUT of less than 10 s was considered abnormal. In testing for TBUT, fluorescein dye was added to the eye and the tear film was evaluated by slit-lamp biomicroscopy with cobalt blue light while the patient avoided blinking until hypofluorescent spots appeared.

During the Schirmer I test, a wetting of less than 10 mm after 5 min without anaesthesia was considered abnormal. The filter paper was folded 5 mm from one end and the folded tip of the filter paper was applied to the lower eyelid margin at the junction of the middle and outer thirds of the lower lid, being careful not to touch the cornea or lashes. The patients were asked to keep their eyes closed during the test.

To examine the contrast sensitivity, we used two boards, one for each eye. The letter plate was shown at a distance of 1 m from the eye and the contrast was assessed from the strongest in the upper left to the weakest in the lower left. 

According to the total number of letters identified, the sensitivity was classified as follows:

- Normal contrast sensitivity—the patient can read 33 letters or more;
- Subnormal sensitivity—the patient can read between 27 and 32 letters;
- Poor sensitivity—the patient can read less than 26 letters;
- 0 sensitivity—the patient cannot read anything.

The inclusion criteria were as follows: adult male or female diagnosed with DED, who completed the Functioning Questionnaire—25 (VFQ-25) version 2000, modified and adapted for this research, when they were included in the study and after receiving different types of treatment, and the follow-up period being at least 1 year. All the patients underwent medical treatment (tear secretion substitutes in the form of artificial tear drops, ointments, conjunctival inserts, autologous serum, mucolytics-acetylcysteine, sodium hyaluronate, and local and general non-steroidal or steroidal anti-inflammatory drugs applied in times of crisis) or invasive procedures (temporary or permanent occlusion of the lacrimal punctum; in severe cases, temporary tarsorrhaphy was recommended). All 121 patients received medical treatment for DED, 46 needed occlusion of the lacrimal punctum, and in 3 cases, temporary tarsorrhaphy was performed.

The exclusion criteria were as follows: patients who declined to participate in the study, who refused to sign the informed consent or to fill out the questionnaire, who refused the complete ophthalmological examination, who recently underwent ophthalmological surgery, or who had other ophthalmological pathologies such as cataract or age-related macular degeneration. 

Initially, the study included 348 participants diagnosed with DED. Due to the fact that not all participants completed the questionnaire a second time, and due to the other exclusion criteria, only 121 participants remained in the research.

We advised that all patients lower the temperature in the room, humidify the air, and wear glasses that encompass the eyes creating a seal around them in order to prevent tear evaporation.

For this study, we used the Visual Functioning Questionnaire—25 (VFQ-25) version 2000, modified and adapted for our research, which included 19 questions. All the patients were asked to complete this questionnaire before and after the treatment. Participants were asked to complete all questions without missing any data. The questionnaire score was calculated for each patient. The score ranged from 0 to 100, a lower score indicating more limitations in daily activities and a socio-cultural impact.

Validation of the questionnaire: Pre-treatment, the item intercorrelation matrix provides a picture of the degree of association between items. The values are useful to show that there are no problems constructing the respective items and there is not a high degree of similarity. Cronbach’s alpha = 0.879 is a good value in relation to the required level of 0.700 to validate the application of this questionnaire. 

## 3. Results

The number of patients initially included in the study was 348, but after applying the exclusion criteria, only 121 remained in our research (Table 1).

### 3.1. Item I1: Difficulties in Reading a Newspaper Text

The answers to this question revealed that patients have high (23.1%) and very high (70.2%) difficulty reading a newspaper text. Pre-treatment, the proportion of patients with high and very high difficulties was slightly higher in women over 70 years of age (*p* > 0.05) (Table 2). 

Post-treatment, only 15.3% of patients with very high difficulty and 28.6% of those with high difficulty still had minimal difficulty reading a newspaper text, but the percentage distributions did not show statistically significant differences (*p* = 0.223) (Table 2).

The assessment of contrast sensitivity in relation to the difficulty of reading a newspaper text shows a significantly higher average value (147.29 vs. 121.18; *p* = 0.046) at the spatial frequency of 6 cycles/grade, after which normalization begins.

### 3.2. Item I2: Recognition of People Met

After studying the case reports, it was noted that 61.2% of the surveyed patients had very high difficulties and 25.6% had high difficulties in recognizing the people they already met, slightly more frequent in females and in people over 70 years old (*p* > 0.05) (Table 3). 

Post-treatment, only 6.8% of patients with very high difficulty and 12.9% of those with high difficulty still had minimal difficulty recognizing encounters, but the percentage distributions were insignificant (*p* = 0.444) (Table 3).

**Figure 1:**
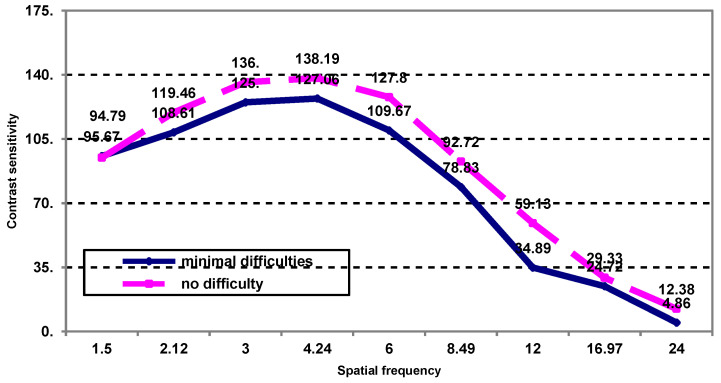
The contrast sensitivity as a function of spatial frequency in patients who have minimal post-treatment visual difficulties recognizing a person.

The assessment of contrast sensitivity as a function of spatial frequency in patients with minimal post-treatment difficulty recognizing a person shows no significant differences (Figure 1). 

### 3.3. Item I3: Difficulties in Reading Prices

Pre-treatment, 73.6% of patients surveyed had very high difficulty and 17.4% had high difficulty reading product prices when shopping. The proportion of patients with very high reading difficulties was slightly higher in females and in the age group over 70 years (*p* > 0.05). Only one female patient aged over 70 years reported no pre-treatment difficulty reading prices when shopping, which was maintained post-treatment (Table 4). 

Post-treatment, minimal difficulty in reading prices when shopping persisted in 40.4% of patients with very high difficulty and in 28.6% of those with high difficulty, showing statistically significant percentage differences (*p* = 0.05) (Table 4).

**Figure 2:**
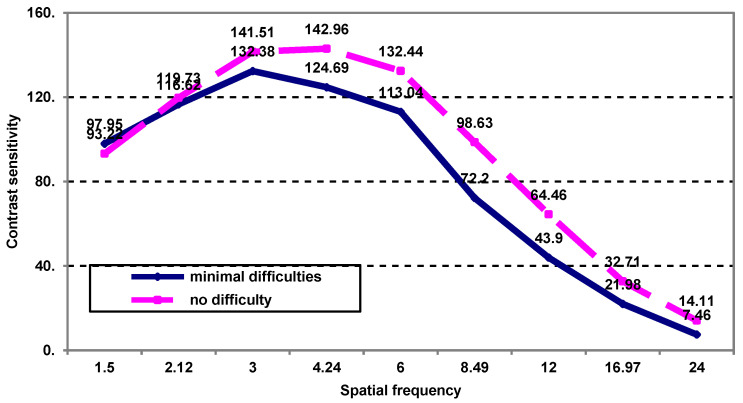
The contrast sensitivity as a function of spatial frequency in patients who have minimal post-treatment visual difficulties in reading prices.

The evaluation of post-treatment contrast sensitivity by spatial frequency in patients with minimal difficulty reading shopping prices highlights the following (Figure 2):

- Up to a spatial frequency of 4.24 cycles/grade, there are increases in average contrast sensitivity values with no significant differences in patients with minimal visual difficulties in reading prices compared to those who do not report such difficulties;
- At a spatial frequency of 4.24 cycles/grade, there is a significantly lower average contrast sensitivity value in patients with minimal difficulty reading shopping prices (124.69 vs 142.96; *p* = 0.026);
- After this frequency, the regression of contrast sensitivity begins, but the average level is significantly lower in patients with visual reading difficulties (*p* < 0.05).

### 3.4. Item I4: Vision Difficulties When Walking on Uneven Ground

Pre-treatment, when walking on uneven ground, 62.8% of the surveyed patients had very high visual difficulties and 25.6% had high visual difficulties, while 1.7% of them reported no visual difficulties when walking on uneven ground. The proportion of patients with high and very high visual difficulties when walking on uneven ground was slightly greater in females over 70 years of age (*p* > 0.05). Two patients, one male and one female, aged over 70 reported no pre-treatment visual difficulties when walking on uneven ground, which was maintained post-treatment (Table 5). 

Post-treatment, minimal difficulty when moving on uneven terrain due to vision persisted in 7.9% of patients with very high difficulty and 9.7% of those with high difficulty, but the percentage differences were statistically insignificant (*p* = 0.965) (Table 5). 

Post-treatment, the assessment of contrast sensitivity as a function of spatial frequency in patients with minimal difficulty walking on uneven ground shows no significant percentage differences. 

### 3.5. Item I5: Visual Difficulties during Manual Work

Before treatment, 66.9% of patients surveyed had very high difficulty and 26.4% had high difficulty in seeing during manual work. Without showing significant percentage differences, the proportion of patients with visual difficulties during manual work was slightly higher in women over 70 years of age (*p* > 0.05) (Table 6). 

After treatment, minimal visual difficulties still persisted during manual work in 14.8% of patients with very high difficulties and in 9.7% of those with high difficulties, but the percentage differences were not statistically significant (*p* = 0.399) (Table 6).

**Figure 3:**
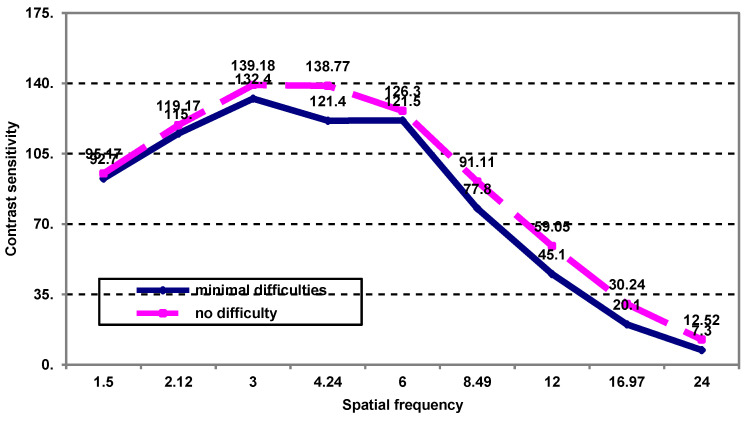
The contrast sensitivity as a function of spatial frequency in patients who have minimal post-treatment visual difficulties during manual work.

On the studied case series, there were no significant differences between the average contrast sensitivity values post-treatment, according to spatial frequency, in patients with minimal difficulties during manual work (Figure 3).

### 3.6. Item I6: Difficulties Reading a Text on TV

The answers to this question revealed that, before treatment, patients had high difficulty (28.9%) and very high difficulty (63.6%) reading a text on TV. 

The proportion of patients with very high difficulty reading a text on TV was slightly more significant in women and in patients over 70 years of age (*p* > 0.05) (Table 7). 

Post-treatment, only 13% of patients with very high difficulty and 5.7% of those with high difficulty still had minimal difficulty reading a text on TV, which are statistically insignificant percentage distributions (*p* = 0.223) (Table 7). 

The average values of contrast sensitivity as a function of spatial frequency show no significant differences in patients with difficulties reading a text on TV compared to those without such difficulties. 

### 3.7. Item I7: Vision Difficulties during Favorite Activities

Pre-treatment, 43.4% of the surveyed patients had very high difficulty and 47.5% had high difficulty in seeing when performing their favorite activities.

The following differences can be noted when practicing hobbies (Table 8):

- Men report very high visual difficulties (54.7%), while women report high difficulties (61.4%) (*p* = 0.044);
- The proportion of patients over 70 years of age with high and very high visual difficulties while performing their favorite activities was slightly higher when compared to patients under 70 years of age (*p* = 0.881).

Post-treatment, minimal visual difficulties still persisted during the practice of favorite activities in 3.8% of patients with very high difficulties and 1.8% of those with high difficulties, but the percentage differences were not statistically significant (*p* = 0.856) (Table 8). 

**Figure 4:**
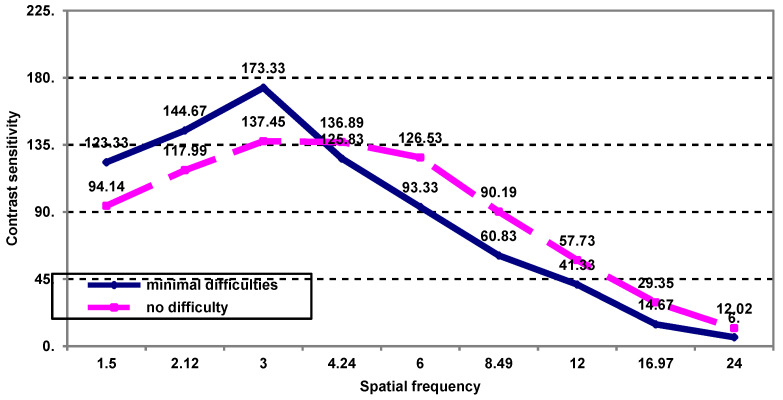
The contrast sensitivity as a function of spatial frequency in patients who have minimal post-treatment difficulties during their favorite activities.

Assessment of post-treatment contrast sensitivity by spatial frequency in patients with minimal difficulty during favorite activities highlights the following (Figure 4):

- Up to the spatial frequency of 3 cycles/degree, there are significantly higher average contrast sensitivity values in patients with minimal visual difficulties compared to those not reporting such difficulties (173.33 vs 137.46; *p* = 0.013);
- After this frequency, there is a regression of average values, the contrast sensitivity being slightly lower in patients with minimal visual difficulties during favorite activities without being statistically significant (*p* > 0.05).

### 3.8. Item I8: Considerations on Visual Difficulties in Daily Activities

Before treatment, 51.2% of the surveyed patients had very high visual difficulties and 40.5% had high visual difficulties in daily activities, slightly more frequent in women and in patients over 70 years of age (*p* > 0.05) (Table 9). 

After treatment, only 22.6% of patients with very high difficulties and 6.1% of those with high difficulties still had minimal vision difficulties in daily activities, but the percentage distributions were not statistically significant (*p* = 0.056) (Table 9). 

The average values of contrast sensitivity as a function of spatial frequency show no significant differences in patients with visual difficulties in daily activities compared to those without such difficulties post-treatment.

### 3.9. Item I9: Degree of Satisfaction with Seeing

Only 5% of patients responding to the questionnaire were satisfied or very satisfied with their vision, while 59.5% were very dissatisfied.

Using a quantitative transformation algorithm, a score was calculated for items I1–I8 to assess the degree of difficulty perceived by the patient before treatment. 

The scores ranged from 7, indicating only minimal visual difficulties, to 24, indicating a high degree of visual difficulty, with a moderate variance in the series of values (20%). The average scores showed a value of 20.13 ± 3.97, which classifies the study group as having a high perception of vision difficulty, with no significant differences between gender (*p* = 0.571) or age groups (*p* = 0.275) (Table 10). 

The response rating score according to perceived degree of visual difficulty and the degree of satisfaction evoked by patients are significantly correlated (*p* = 0.001), with patients who reported being dissatisfied or very dissatisfied having a significantly higher average score compared to other patients (Table 10).

Post-treatment, all patients in the analyzed group were satisfied or very satisfied with their visual acuity.

The post-treatment calculated score shows a significant improvement in the degree of visual difficulty (*p* = 0.001); patients’ perception shows that they no longer have visual difficulties in most cases (Table 11). 

Post-treatment, the average values of contrast sensitivity recorded by degree of satisfaction show no significant differences.

## 4. Discussion

Dry eye disease is a condition typically caused by tear dysfunction, a quantitative or qualitative insufficiency of tear film, and is more recently defined as a disease of the ocular surface. Dry eye syndrome is the most commonly faced problem in general ophthalmology; it is mainly caused by the quality of tears secreted by the tear glands [13]. 

The tear film has three layers: lipidic, aqueous, and mucous. The constituents are complex, with as many as a hundred distinct proteins identified. One of the causes for this syndrome is the ageing process, due to a decrease in the lipid concentration of tears, and it is more common in women. Other factors include climate, air conditioning, cigarette smoke, computers, thyroid disease, depression, or psychiatric medications [13].

Possible systemic conditions associated with DED include metabolic diseases such as thyroid disease, diabetes mellitus and hyperlipidemia, cardiovascular diseases such as ischemic heart disease, cardiac arrhythmias, peripheral vascular disorders, stroke, immunologic pathologies, degenerative diseases such as arthritis, mental conditions, and malignancies [14].

Quality of life analyses are particularly useful for medical practice in assessing the physical, psychological, and social effects of this illness and medical treatment on people’s daily lives, in analyzing the effects of treatment or illness from the patient’s point of view, and in determining the patient’s needs for psychological, physical, and social support during this time.

Numerous studies used Visual Function Questionnaire (VFQ-25) in order to measure the self-reported vision-targeted health status that are most important for persons who have chronic eye diseases [15,16,17]. Cronbach’s alpha is a statistic frequently cited by authors to show that tests and scales that have been used for research projects are appropriate to the purpose. Authors usually cite alpha values with few comments to explain why they find this statistic relevant and rarely interpret the result for readers beyond quoting an arbitrary threshold for a valid value. This was typically seen as ≥0.70 or >0.70, while other authors referred more vaguely to the acceptable values of 0.7 or 0.6 [18]. In the current research, Cronbach’s alpha = 0.879, which represents a good value in relation to the required level of 0.700 to validate the application of this questionnaire.

Dry eye disease is more common in women than in men and has a higher prevalence with age [19]. The prevalence of dry eye syndrome varies from approximately 5 to 50% in population-based studies. Approximately 1 out of 7 individuals with ages between 65 and 84 years old report symptoms of dry eye frequently [20].

Various epidemiological studies suggested that dry eye prevalence increases in women and men every five years after the age of 50, with higher prevalence in women compared to men [21,22,23,24,25,26]. Age and female sex have been found to represent the major risk factors for dry eye. These findings are supported by the reduced tear production in women through the sixth decade of life [27,28,29]. These results are similar with the ones found in the current study where the dry eye syndrome was discovered more commonly after the age of 70, with 65% of cases being women. 

A study conducted in Singapore showed that symptomatic dry eye disease was associated with difficulties in realizing different types of vision-related daily activities [30]. Other studies showed that patients with symptomatic dry eye disease were having challenges with reading newspapers and road signs, facial recognition of their friends, watching TV, cooking, using a computer, and driving during the night [22,31]. The findings from this research are similar to the ones from these studies. The results showed that before treatment, patients have high (23.1%) and very high (70.2%) difficulty reading a newspaper text, 61.2% of the surveyed patients have very great difficulties in recognizing the people they met, and 73.6% of patients surveyed had very high difficulty reading prices when shopping. Moreover, in the current study, we found that when walking on uneven ground, 62.8% of the surveyed patients had very high visual difficulties, 25.6% had high visual difficulties, while 1.7% of them reported no visual difficulties. The results showed that 66.9% of patients surveyed have very high difficulty and 26.4% have high difficulty in seeing during manual work. Before treatment, the patients from the current study have high difficulty (28.9%) and very high difficulty (63.6%) reading a text on TV and 43.4% of them have very high difficulty in seeing when performing their favorite activities.

Difficulties in realizing the previously mentioned tasks may be associated with long-term gazing and diminished blinking secondary to greater visual requirements in performing these activities. Moreover, living in an environment with air conditioning and low humidity can lead to instability and high evaporation of the tear film, which can determine irregularity of the optical refracting surfaces [30].

Various studies reported an important decrease in the blinking rate associated with digital screen devices work, reading, and increased driving speed, which can determine tear film instability [32,33]. A research study conducted by Li et al. showed that vision-related quality of life in patients with DED was impaired and was correlated with anxiety and depression [34].

The findings from this research showed that after receiving different types of treatment for dry eye disease such as temporary or permanent lacrimal point occlusion or drug treatment consisting of administration of tear secretion substitutes in the form of artificial tear drops, ointments, conjunctival inserts, or autologous serum, visual difficulties improved in the majority of cases. 

Therefore, post-treatment, only 15.3% of patients with very high difficulty still had minimal problems reading a newspaper text, only 6.8% of patients from the same group still had minimal problems recognizing encounters, and minimal difficulty in reading prices when shopping persisted in 40.4% of patients with very high difficulty. Moreover, the results from this study showed that in the group with very high difficulty, after treatment, difficulty in moving over uneven terrain due to vision persisted in 7.9% of patients, visual difficulties still persisted during manual work in 14.8% of patients, and only 13% of patients with very high difficulty still had minimal problems reading a text on TV. Regarding favorite activities, the results from the current research showed that post-treatment visual difficulties still persisted in 3.8% of patients with very high difficulties. 

Various studies showed that the quality of life improves after different types of treatment in patients with dry eye disease [35,36] and these findings are similar with the ones mentioned in this study.

A study conducted in the Northern European population showed that sleep quality was significantly reduced in participants with dry eye disease, even after correcting for comorbidities [37]. A study from 2022 researched the association between DED symptoms and daily beverage intake, but no significant association was found [38]. In the current paper, we did not investigate these aspects. Further studies are needed to examine this association.

A study conducted by Szczotka-Flynn et al. on the impact of dry eye disease on visual acuity and contrast sensitivity concluded that low visual acuity, rather than worse contrast sensitivity, leads to visual symptoms in DED [39]. Another research study about contrast sensitivity in patients with DED concluded that contrast sensitivity was not significantly different across subject groups [40]. In the present study, the assessment of contrast sensitivity in relation to the difficulty of reading a newspaper text showed a significantly higher average value (147.29 vs. 121.18; *p* = 0.046) at the spatial frequency of 6 cycles/grade, after which normalization begins. On the other hand, the assessment of contrast sensitivity as a function of spatial frequency in patients with minimal post-treatment difficulty recognizing a person or in patients with minimal difficulty walking on uneven ground showed no significant differences.

The main strength of this research is that it addresses an important pathology, which is becoming more common in both adults and children. We evaluated the most frequent associations between DED and vision-related daily activities.

One of the main weaknesses of this study is that we did not separate the answers to the questionnaires according to the treatment given to the patients and the same questions were used regardless of the type of treatment. Another limitation of this study is represented by the fact that almost all patients included were old and many of them may experience difficulties in performing daily activities with visual and physical problems with or without dry eye disease.

Relevant information about self-reported vision-targeted health status was obtained through self-answered questionnaires, which are not always reliable. Further larger-scale studies are needed to address the effects of DED on performing daily activities.

## 5. Conclusions

In the present research, prior to treatment, patients had very high difficulty reading a text in a newspaper or on TV, reading prices in a shop, recognizing people they met, walking on uneven ground, and performing manual work, favorite activities, and actions of everyday life. 

Post-treatment, visual difficulties improved in the majority of cases and all patients were satisfied or very satisfied with the acquired visual acuity, regardless of the treatment method. 

In conclusion, we found that symptomatic dry eye disease was associated with reduced ability in performing several important vision-related daily tasks and has a significant impact on the quality of life and visual performance.

## Figures and Tables

**Table 1:** Distribution of patients by age group before and after the exclusion criteria.

Age Group	Patients Who Underwent Invasive Procedures			Patients Who Underwent Drug Treatment			Total		
	N	n	%	N	n	%	N	N	%
<50 years	40	9	22.5	49	12	24.5	89	21	23.6
60–69 years	21	6	28.6	49	17	34.7	70	23	32.9
70–79 years	41	22	53.7	96	18	18.8	137	40	29.2
≥80 years	15	12	80.0	37	25	67.6	52	37	71.2
Total	117	49	41.9	231	72	31.2	348	121	34.8

N = number of patients included initially in the study; n = number of patients who remained in the study after exclusion criteria. The mean TBUT was 3.2 ± 1.3 s and Schirmer I test was 7.4 ± 6.2 mm/5 min.

**Table 2:** The percentage distribution of patients with reading difficulties of a newspaper text according to pre- and post-treatment study group characteristics.

Characteristics	Very High Difficulty		High Difficulty		Minimal Difficulty		No Difficulty		*p* Values
	n	%	n	%	n	%	N	%	
**Pre-treatment**	85	70.2	28	23.1	7	5.7	1	0.8	-
**Gender**									0.253
Male	42	49.4	9	32.1	4	57.1	1	100.0	
Female	43	50.6	19	67.9	3	42.9	-	-	
**Age group**									0.396
<70 years	38	44.7	8	28.6	3	42.9	-	-	
70+ years	47	55.3	20	71.4	4	57.1	1	100.0	
**Post-treatment**									0.223
Minimal difficulty	13	15.3	8	28.6	-	-	-	-	
No difficulty	72	84.7	20	71.4	7	100.0	1	100.0	

**Table 3:** The percentage distribution of patients with difficulty in recognizing a person they met according to pre- and post-treatment study group characteristics.

Characteristics	Very High Difficulty		High Difficulty		Minimal Difficulty		No Difficulty		*p* Values
	n	%	N	%	n	%	n	%	
**Pre-treatment**	84	61.2	31	25.6	14	11.6	2	1.7	-
**Gender**									0.700
Male	35	47.3%	12	38.7%	8	57.1%	1	50.0%	
Female	39	52.7%	19	61.3%	6	42.9%	1	50.0%	
**Age group**									0.958
<70 years	31	41.9%	12	38.7%	5	35.7%	1	50.0%	
70+ years	43	58.1%	19	61.3%	9	64.3%	1	50.0%	
**Post-treatment**									0.444
Minimal difficulty	5	6.8	4	12.9	-	-	-	-	
No difficulty	69	93.2	27	87.1	14	100.0	2	100.0	

**Table 4:** The percentage distribution of patients with reading difficulties reading prices according to pre- and post-treatment study group characteristics.

Characteristics	Very High Difficulty		High Difficulty		Minimal Difficulty		No Difficulty		*p* Values
	n	%	N	%	n	%	N	%	
**Pre-treatment**	89	73.	21	17.4	8	8.3	1	0.8	-
**Gender**									0.178
Male	37	41.6	12	57.1	7	70.0	-	-	
Female	52	58.4	9	42.9	3	30.0	1	100.0	
**Age group**									0.410
<70 years	39	43.8	8	38.1	2	20.0	-	-	
70+ years	50	56.2	13	61.9	8	80.0	1	100.0	
**Post-treatment**									**0.050**
Minimal difficulty	36	40.4%	6	28.6%	-	-	-	-	
No difficulty	53	59.6%	15	71.4%	10	100.0%	1	100.0%	

**Table 5:** The percentage distribution of patients with vision difficulties walking on uneven ground according to pre- and post-treatment study group characteristics.

Characteristics	Very High Difficulty		High Difficulty		Minimal Difficulty		No Difficulty		*p* Values
	n	%	n	%	n	%	n	%	
**Pre-treatment**	76	62.8	31	25.6	12	9.9	2	1.7	-
**Gender**									0.209
Male	32	42.1%	14	45.2%	9	75.0%	1	50.0%	
Female	44	57.9%	17	54.8%	3	25.0%	1	50.0%	
**Age group**									0.631
<70 years	32	42.1%	13	41.9%	4	33.3%	-	-	
70+ years	44	57.9%	18	58.1%	8	66.7%	2	100.0%	
**Post-treatment**									0.965
Minimal difficulty	6	7.9%	3	9.7%	1	8.3%	-	-	
No difficulty	70	92.1%	28	90.3%	11	91.7%	2	100.0%	

**Table 6:** The percentage distribution of patients with visual difficulties during manual work according to pre- and post-treatment study group characteristics.

Characteristics	Very High Difficulty		High Difficulty		Minimal Difficulty		No Difficulty		*p* Values
	n	%	n	%	n	%	n	%	
**Pre-treatment**	81	66.9	32	26.4	8	6.6	-	-	-
**Gender**									0.358
Male	34	42.0	17	53.1	5	62.5	-	-	
Female	47	58.0	15	46.9	3	37.5	-	-	
**Age group**									0.647
<70 years	34	42.0	13	40.6	2	25.0	-	-	
70+ years	47	58.0	19	59.4	6	75.0	-	-	
**Post-treatment**									0.399
Minimal difficulty	12	14.8	3	9.4	-	-	-	-	
No difficulty	69	85.2	29	90.6	8	100.0	-	-	

**Table 7:** The percentage distribution of patients with reading difficulties of a text on TV according to pre- and post-treatment study group characteristics.

Characteristics	Very High Difficulty		High Difficulty		Minimal Difficulty		No Difficulty		*p* Values
	n	%	n	%	n	%	n	%	
**Pre-treatment**	77	63.6	35	28.9	9	7.4	-	-	-
**Gender**									0.606
Male	35	45.5	18	51.4	3	33.3	-	-	
Female	42	54.5	17	48.6	6	66.7	-	-	
**Age group**									0.554
<70 years	34	44.2	12	34.3	3	33.3	-	-	
70+ years	43	55.8	23	65.7	6	66.7	-	-	
**Post-treatment**									0.233
Minimal difficulty	10	13.0%	2	5.7%	-	-	-	-	
No difficulty	67	87.0%	33	94.3%	9	100.0%	-	-	

**Table 8:** The percentage distribution of patients with difficulties in seeing during hobbies according to pre- and post-treatment study group characteristics.

Characteristics	Very High Difficulty		High Difficulty		Minimal Difficulty		No Difficulty		*p* Values
	n	%	n	%	n	%	n	%	
**Pre-treatment**	53	43.4	58	47.5	7	5,7	4	3.3	-
**Gender**									**0.044**
Male	29	54.7%	22	38.6%	5	71.4%	-	-	
Female	24	45.3%	35	61.4%	2	28.6%	4	100.0%	
**Age group**									0.881
<70 years	23	43.4%	21	36.8%	3	42.9%	2	50.0%	
70+ years	30	56.6%	36	63.2%	4	57.1%	2	50.0%	
**Post-treatment**									0.856
Minimal difficulty	2	3.8%	1	1.8%	-	-	-	-	
No difficulty	51	96.2%	56	98.2%	7	100.0%	4	100.0%	

**Table 9:** The percentage distribution of patients with visual difficulties in daily activities according to pre- and post-treatment study group characteristics.

Characteristics	Very High Difficulty		High Difficulty		Minimal Difficulty		No Difficulty		*p* Values
	n	%	n	%	n	%	n	%	
**Pre-treatment**	62	63.6	49	28.9	10	7.4	-	-	-
**Gender**									**0.044**
Male	28	45.2%	22	44.9%	6	60.0%	-	-	
Female	34	54.8%	27	55.1%	4	40.0%	-	-	
**Age group**									0.881
<70 years	27	43.5%	18	36.7%	4	40.0%	-	-	
70+ years	35	56.5%	31	63.3%	6	60.0%	-	-	
**Post-treatment**									0.056
Minimal difficulty	14	22.6%	3	6.1%	2	20.0%	-	-	
No difficulty	48	77.4%	46	93.9%	8	80.0%	-	-	

**Table 10:** Statistical indicators of the vision difficulty assessment score.

Characteristics	N	Average	Standard Deviation	Standard Error	Confidence Range		Min	Max	*p* Value Test F
					−95% CI	+95% CI			
**Gender**									
Male	56	19.91	4.55	0.609	18.69	21.13	7	24	0.571
Female	65	20.32	3.42	0.424	19.48	21.17	7	24	
Total	121	20.13	3.97	0.361	19.42	20.85	7	24	
**Age group**									
<70 years	49	20.61	3.61	0.516	19.58	21.65	8	24	0.275
70+ years	72	19.81	4.20	0.494	18.82	20.79	7	24	
Total	121	20.13	3.97	0.361	19.42	20.85	7	24	
**Degree of satisfaction (pre-treatment)**									
Very dissatisfied	72	21.72	2.86	0.338	21.05	22.40	9	24	**0.001**
Dissatisfied	43	18.44	3.76	0.574	17.28	19.60	7	24	
Satisfied	3	10.33	4.93	2.848	−1.92	22.59	7	16	
Very satisfied	3	16.00	3.46	2.000	7.39	24.61	14	20	
Total	121	20.13	3.97	0.361	19.42	20.85	7	24	

**Table 11:** Statistical indicators of the assessment score of the degree of pre- and post-treatment vision difficulty.

Score	N	Average	Standard Deviation	Standard Error	Confidence Range		Min	Max	*p* Value Test F
					−95% CI	+95% CI			
**Pre-treatment**	121	20.13	3.973	0.361	19.42	20.85	7	24	**0.001**
**Post-treatment**	121	1.08	1.333	0.121	0.84	1.32	0	6	

## Data Availability

The datasets used and analyzed during the current study are available from the corresponding author on reasonable request.
